# Temperature-Dependent and Threshold Behavior of Sm^3+^ Ions on Fluorescence Properties of Lithium Niobate Single Crystals

**DOI:** 10.3390/ma11102058

**Published:** 2018-10-22

**Authors:** Mingming Yang, Siwei Long, Xin Yang, Shaopeng Lin, Yunzhong Zhu, Decai Ma, Biao Wang

**Affiliations:** 1 School of Physics, Sun Yat-sen University, Guangzhou 510275, China; ymming@mail2.sysu.edu.cn (M.Y.); longsw3@mail.sysu.edu.cn (S.L.); yangx235@mail2.sysu.edu.cn (X.Y.); lshpeng@mail.sysu.edu.cn (S.L.); zhuyzh7@mail.sysu.edu.cn (Y.Z.); 2 Sino French Institute of Nuclear Engineering and Technology, Sun Yat-sen University, Zhuhai 519082, China; 3 School of Physics Science & Engineering, Institute of Optoelectronic and Functional Composite Materials, State Key Lab Optoelectronic Materials and Technologies, Sun Yat Sen University, Guangzhou 510275, China

**Keywords:** rare earth-doped materials, fluorescence spectroscopy, lithium niobate, threshold concentration, temperature, J-O theory

## Abstract

Temperature-dependent and threshold behavior of Sm^3+^ ions on fluorescence properties of lithium niobate (LiNbO_3_, LN) single crystals were systematically investigated. The test materials, congruent LiNbO_3_ single crystals (Sm:LN), with various concentrations of doped Sm^3+^ ions from 0.2 to 2.0 mol.%, were grown using the Czochralski technique. Absorption spectra were obtained at room temperature, and photoluminescence spectra were measured at various temperatures in the range from 73 K to 423 K. Judd–Ofelt theory was applied to calculate the intensity parameters Ω_t_ (t = 2, 4, 6) for 1.0 mol.% Sm^3+^-doped LiNbO_3_, as well as the radiative transition rate, *A*_r_, branching ratio, *β*, and radiative lifetime, *τ*_r_, of the fluorescent ^4^G_5/2_ level. Under 409 nm laser excitation, the photoluminescence spectra of the visible fluorescence of Sm^3+^ mainly contains 568, 610, and 651 nm emission spectra, corresponding to the energy level transitions of ^4^G_5/2_→^6^H_5/2_, ^4^G_5/2_→^6^H_7/2_, and ^4^G_5/2_→^6^H_9/2_, respectively. The concentration of Sm^3+^ ions has great impact on the fluorescence intensity. The luminescence intensity of Sm (1.0 mol.%):LN is about ten times as against Sm (0.2 mol.%):LN at 610 nm. The intensity of the fluorescence spectra were found to be highly depend on temperature, as well as the concentration of Sm^3+^ ions in LiNbO_3_ single crystals, as predicted; however, the lifetime changed little with the temperature, indicating that the temperature has little effect on it, in Sm:LN single crystals. Sm:LN single crystals, with orange-red emission spectra, can be used as the active material in new light sources, fluorescent display devices, UV-sensors, and visible lasers.

## 1. Introduction

Lithium niobate (LiNbO_3_, LN), as a full-feature crystal, possesses many excellent properties, such as electro-optical, nonlinear optical, and acousto-optical properties, that enable it to be widely used in electro-optic (EO) signal modulation, lasers, biological imaging, temperature sensors, and many other examples. In recent years, the optical properties of rare earth (RE)^3+^-doped LN single crystals, such as Nd^3+^, Er^3+^, Ho^3+^, Yb^3+^, Pr^3+^, and Dy^3+^:LN, have been studied in detail [1,2,3,4,5,6,7,8,9]; some of these crystals have been widely applied in solid-state optical devices. The trivalent samarium ion (Sm^3+^), which has a complex energy level structure, exhibits emission in the visible region, and is known for its rich orange emission at 610 nm and red emission 651 nm, both of which originate from the ^4^G_5/2_ state; such an emission is interesting for many photonic applications. For example, the reddish-orange emission band in the visible region, corresponding to the emission spectra of the ^4^G_5/2_→^6^H_7/2_ and ^4^G_5/2_→^6^H_9/2_ transitions, is desirable for uses in high-density optical storage, color displays, medical diagnostics, fluorescent display devices, and sensors [2,3,10,11]. More specifically, Sm^3+^-containing hosts have been offered as infrared counters, as an activated solid, and as a means to increase the photovoltaic solar cell conversion efficiency via down-conversion of the solar spectrum [12,13].

However, most of those reports have mainly focused on fluorosilicate glasses [14], alkyl-barium-bismuth-tellurite (LKBBT) glass [15], and other glasses at room temperature [16]. Dominiak-Dzik [2] reported the spectroscopic properties of a Sm:LN single crystal with a fixed Sm concentration at 0.65 wt.%, while Wang [3] also studied the optical properties of Mg:Sm (0.3 at%):LN and Zn:Sm (0.3 at%):LN at room temperature. Obviously, the doping concentration of rare metal ions has a strong impact on the fluorescence intensity of the doped materials [4]. Moreover, temperature also influences the luminescence property, such as Sm^3+^-doped oxyfluoride powders [17].

In this paper, all the congruent Sm:LN single crystals were grown using the Czochralski method. Our work is mainly focused on the impact of temperature and Sm^3+^-doped concentration on the spectral emission properties of Sm:LN single crystals. The effect of temperature and Sm^3+^-doped concentration variation, on the emission properties of Sm^3+^-doped LN single crystals, was investigated systematically. To predict the radiative properties, such as the radiative transition probabilities, radiative lifetimes, and branching ratios, a detailed J-O analysis was conducted. The X-ray diffraction (XRD) data and ultraviolet–visible (UV-vis) absorption spectra were used to qualitatively analyze the occupation mechanism of Sm^3+^ ions in the crystal lattice. For each specimen, we also calculated the stimulated emission cross-section *σ_em_*, which is an important parameter signifying the energy extraction rate from a lasing material.

## 2. Materials and Methods

**Figure 1:**
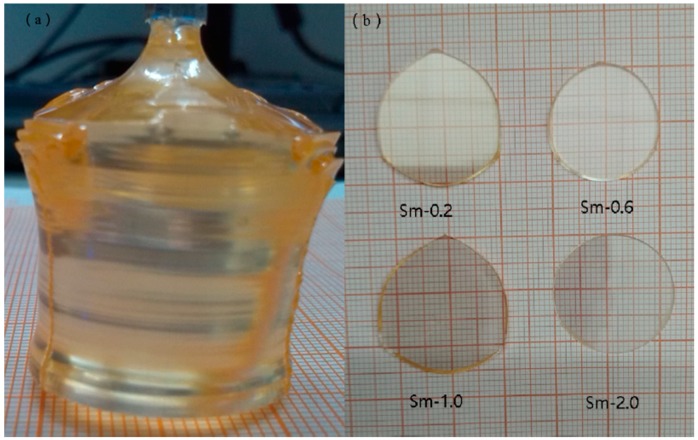
(**a**) As-grown and homogeneous single crystals orange 1.0 mol.% Sm^3+^-doped lithium niobate (LN) with the diameter of 25 mm and the length of 35 mm. (**b**) The double-side polished sample wafers, with Sm^3+^-doped concentration from 0.2 mol.% to 2.0 mol.%, were marked as Sm-0.2, Sm-0.6, Sm-1.0, and Sm-2.0, respectively.

LiNbO_3_ single crystals doped with Sm_2_O_3_ were grown using the Czochralski method, along the *c*-axis, from a congruent melt composition (Li/Nb = 48.6/51.4). The concentrations of Sm^3+^ in the melt were 0.2, 0.6, 1.0, and 2.0 mol.%. The raw materials were Li_2_CO_3_, Nb_2_O_5_, and Sm_2_O_3_, and the purity of these raw materials were 99.99%. To remove CO_2_, the raw samples were heated to 750 °C slowly, and maintained at that temperature for 3 h, and then heated up to 1150 °C and maintained at that temperature for 3 h to synthesize Sm:LN polycrystals in a Pt crucible. The crystals were grown under the optimum conditions of a rotation rate of 15 rpm and pulling rate of 1.0 mm/h. The as-grown Sm:LN single crystals are transparent, crack-free, and faint yellow, as shown in Figure 1a. The crystals were polarized at 1180 °C with a direct current density of 5 mA/cm^2^. The polarized crystals were cut and double-side polished into *c*-axis wafers (X × Y × Z = 25 × 25 × 2 mm^3^), as shown in Figure 1b.

The crystalline phase was identified by an X-ray diffraction (XRD, D-MAX-2200 VPC) apparatus (HITACHI, Hitachi, Japan) equipped with a copper Kα radiation source. The diffraction data were acquired over scattering angles (2*θ*) from 10° to 90°. The absorption spectra were recorded using a UV–vis–NIR spectrophotometer (UV-3600, SHIMADZU, Kyoto, Japan) at room temperature. We also obtained fluorescence emission spectra at a series of temperatures (73, 173, 298, 373, and 423 K) using a photoluminescence spectrometer (FLS920, Edinburgh, UK) equipped with a temperature controller under 409 nm excitation from a 450 mW Xe lamp. The lifetime of the ^4^G_5/2_ level energy was measured using a μF920 flash lamp, and the induced time-resolved curves were recorded at various temperatures.

## 3. Results

### 3.1. XRD Studies of Sm:LN Single Crystals

**Figure 2:**
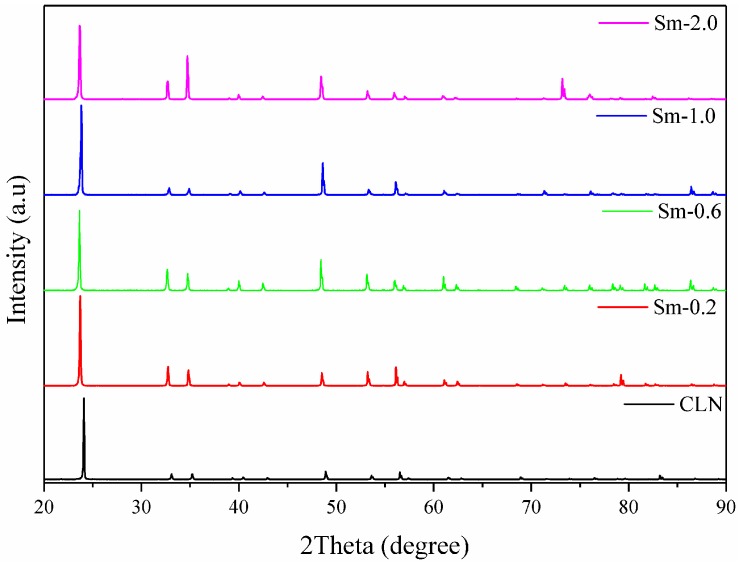
X-ray powder diffraction patterns of congruent LiNbO_3_ and series Sm^3+^-doped LiNbO_3_ single crystals.

As shown in Figure 2, the triclinic phase diffraction peaks of Sm:LN were predominant in the XRD pattern, and are quite similar to the peaks of pure LiNbO_3_. Only the LiNbO_3_ triclinic phase was observed. The results indicate that the doped samarium ion impurity cannot change the lattice structure of LiNbO_3_. The lattice parameters were calculated by Rietveld refinement, and the unit cell volumes were obtained using the formula *V* = (*abc*)·cos30°, as shown in Table 1. With the increasing concentration of doped Sm^3+^ ions, the unit cell volumes of the crystals initially increase. Since the comparable radii of the samarium ion, lithium ion, and niobium ion are 0.964 Å, 0.60 Å, and 0.70 Å, respectively, the doped samarium ions cannot exist as interstitial ions in the crystal.

According to the Li-site vacancy model [18], there are many Li vacancy (V_Li_) sites, and some excessive Nb ions occupy vacant Li sites to form anti-site Nb (${\mathrm{Nb}}_{\mathrm{Li}}^{4+}$) defects, because the Li/Nb ratio is 0.946 in congruent LiNbO_3_. In the Sm-0.2 and Sm-0.6 samples, the weakly doped Sm^3+^ ions replace the anti-site Nb (${\mathrm{Nb}}_{\mathrm{Li}}^{4+}$). In Sm-1.0, all the ${\mathrm{Nb}}_{\mathrm{Li}}^{4+}$ ions are replaced by Sm^3+^ ions; this doping level has exceeded the “threshold value”. As the concentration of Sm^3+^ continues to increase, Sm^3+^ ions may replace both the Li-site and Nb-site, and form ${\mathrm{Sm}}_{\mathrm{Li}}^{2+}$${–\mathrm{Sm}}_{\mathrm{Nb}}^{2-}$ defect centers in the samples. Moreover, the *c*/*a* of Sm:LN lattice decreases as the Sm^3+^ ions concentration increases because the ${\mathrm{Sm}}_{\mathrm{Li}}^{2+}$${–\mathrm{Sm}}_{\mathrm{Nb}}^{2-}$ has a shorter chemical bond.

### 3.2. Absorption Spectra and Judd–Ofelt Analysis

**Figure 3:**
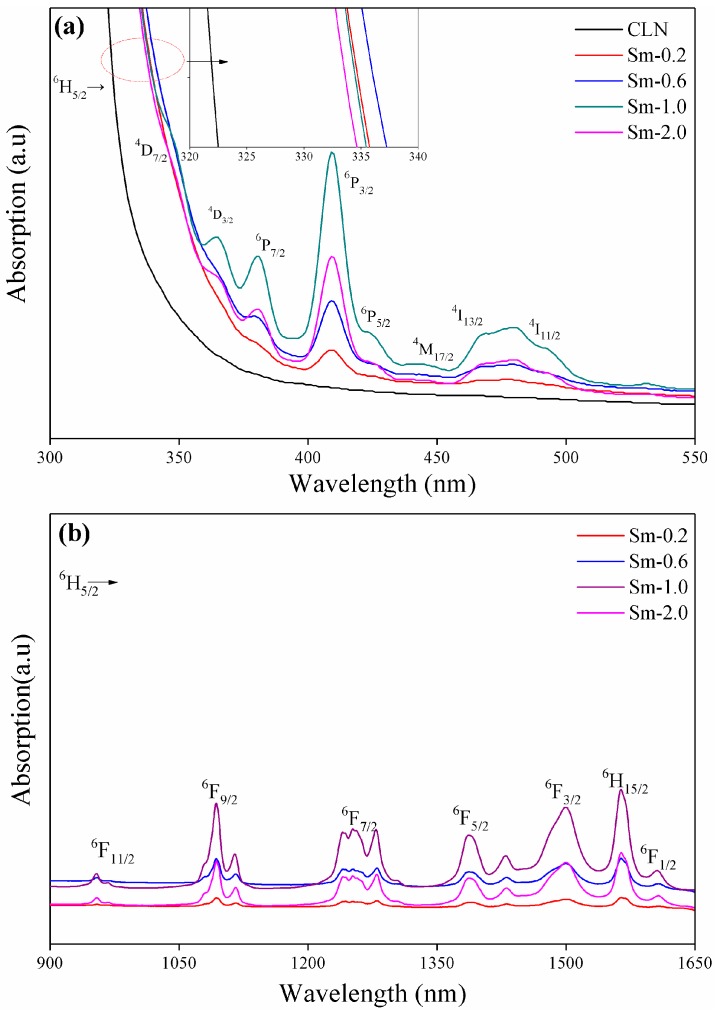
(**a**)UV–vis absorption spectra of Sm:LN single crystals. (**b**) Near-infrared absorption spectra of the Sm:LN single crystals.

The optical absorption spectra of various concentrations of Sm:LN single-crystal samples are shown in Figure 3a for the UV-vis region, and in Figure 3b for the NIR region; these spectra are also related to the transition of Sm^3+^ from the ground state ^6^H_5/2_ to the excited state.

To study the issue of defects and ion occupation in Sm:LN crystals, we calculated the polarizability of the ions by using the equation *F* = (Z*)^2^/*r*, where Z* = *Z* − Σ*s*, and Z*, *Z*, Σ*s*, and *r* represent the effective nuclear charge number, the nuclear charge number, the shield factor, and the radius of the ion, respectively. The calculated values of the polarizability of Li^+^, Nb^5+^, and Sm^3+^ ions were 2.82, 58.51, and 62.31, respectively. The basal optical absorption edge of the lithium niobate crystal is determined by the valence electron transition energy from the 2*p* orbital of O^2−^ to the 4*d* orbital of Nb^5+^ [19]; this absorption edge can be used to analyze the ion occupation issue of the Sm:LN crystal qualitatively.

When the polarizability of the doped ion is lower than that of the replaced ion, the polarizability of O^2−^ decreases, and the transition energy from the 2*p* orbital of O^2−^ to the 4*d* orbital of Nb^5+^ increases. Additionally, the absorption edge shifts to the ultraviolet band and, conversely, the absorption edge shifts to the infrared band [20,21]. Figure 3a shows that the absorption edges of the samples, Sm-0.2 and Sm-0.6, redshift; however, when the concentration reaches 0.6 mol.%, the absorption edge shifts toward the ultraviolet. According to the Li-site vacancy model, when Sm^3+^ ions enter the LiNbO_3_ crystal lattice, they will first replace ${\mathrm{Nb}}_{\mathrm{Li}}^{4+}$ and, then, occupy the normal Li-site. Since the polarization ability is in the order of Sm^3+^ > Nb^5+^ > Li^+^, the transition energy from the 2*p* orbital of O^2−^ to the 4*d* orbital of Nb^5+^ decreases, and results in a shift of the absorption edge to the longer wavelengths when ${\mathrm{Nb}}_{\mathrm{Li}}^{4+}$ defects were replaced by Sm^3+^ ion. After ${\mathrm{Nb}}_{\mathrm{Li}}^{4+}$ are completely replaced by Sm^3+^, the doped ions may replace Li-site and Nb-site to form ${\mathrm{Sm}}_{\mathrm{Li}}^{2+}$${–\mathrm{Sm}}_{\mathrm{Nb}}^{2-}$ center [22,23,24], and give rise to the absorption edge that goes toward the shorter wavelengths; hence, the inversion of the absorption edge illustrates that the threshold concentration of Sm^3+^ in the LiNbO_3_ single crystal is between 0.6 mol.% and 1.0 mol.%. This is due to the interionic distances between O^2−^ and Nb^5+^ becoming expanded with the doped Sm^3+^ concentration, which is caused by the lattice of Sm:LN crystals slightly increasing, as shown in Table 1. The absorption edge will shift to shorter wavelengths when the transition energy from the 2*p* orbital of O^2−^ to the 4*d* orbital of Nb^5+^ decreases. The absorption edge shifts of Sm:LN crystals is different with anti-photorefractive ion, such as Mg^2+^ and Zr^4+^ [25,26], because the polarizability of Mg^2+^ and Zr^4+^ ions is less than that of Nb^5+^, while the polarizability of Sm^3+^ ion is bigger than that of the Nb^5+^ ion.

The J-O theory was independently developed by Judd [27] and Ofelt [28]. We describe some of the relevant and important formulas, as follows.

The calculated oscillator strength of an induced electric dipole transition, from the ground state, Ψ_1_*J*_1_, to the excited state, Ψ_2_*J*_2_, can be given by

(1) $$f_{cal}=\frac{8\pi^{2}mc}{3h\left(2J_{1}+1\right)\lambda }\frac{{\left(n^{2}+2\right)}^{2}}{9n}\sum_{t=2,4,6}\Omega_{t}|\langle \Psi_{1}J_{1}\left\|U^{\left(t\right)}\right\|{\Psi_{2}J_{2}\rangle |}^{2},$$

where *n* is the refractive index of the lithium niobate wafer; 2(*n*^2^ + 2)/9*n* is the Lorentz local field correction and accounts for the dipole–dipole transition; *h* is Planck’s constant; Ω*_t_* (*t* = 2, 4, and 6) denotes the J-O intensity parameters; and ‖U*^(t)^*‖^2^ is the doubly reduced matrix elements of the unit tensor operator [29] evaluated in the intermediate coupling scheme for the transition Ψ_1_*J*_1_→Ψ_2_*J*_2_. The experimental oscillator strength for the transition from the ground state to the excited state (*f_exp_)* is directly proportional to the area under the absorption curve, and is expressed by Equations (2) and (3) [30]:

(2) $$f_{exp}\left(J_{1}\to J_{2}\right)=\frac{{\mathrm{mc}}^{2}}{N\pi e^{2}\lambda^{2}}\int \alpha (\lambda )d\lambda ,$$

(3) $$f_{exp}\left(J_{1}\to J_{2}\right)=\frac{1}{0.43d}\int \mathrm{OD}(\lambda )d(\lambda )=4.318\times 10^{-9}\int \varepsilon (\nu )d\nu ,$$

where *m* and *e* are the mass and charge of the electron, respectively; *c* is the speed of velocity of light; *N* is the concentration of ions doped in the crystal; and *ε*(*υ*) is the molar absorptivity of a band at a wave number, *υ* (cm^−1^). A least-squares fit method is then used for Equation (1) to determine the Ω*_t_* parameters that provide the best fit between the experimental and calculated oscillator strengths. The theoretical oscillator strength, *f_cal_*, is then obtained using Equation (1) and Ω*_t_*.

The root mean square deviation of the fit can be expressed as

(4) $$\delta_{rmse}=\sqrt{\frac{\sum {\left(f_{cal}-f_{exp}\right)}^{2}}{L-3}},$$

where *L* is the number of analyzed groups of the transition bands, and 3 is the number of fitted parameters.

As described above, with the obtained Judd–Ofelt parameters, Ω*_t_*, the radiative properties for the rare earth-doped materials could be evaluated through the relevant expressions given below. The spontaneous emission probability (*A_r_*) from the initial manifold (Ψ_1_*J*_1_) to the final manifold (Ψ_2_*J*_2_) is

(5) $$A_{r}=\frac{64\pi^{4}e^{2}}{3h(2J_{1}+1)\lambda^{3}}\frac{n{\left(n^{2}+2\right)}^{2}}{9}\sum_{t=2,4,6}\Omega_{t}|\langle \Psi_{1}J_{1}\left\|U^{\left(t\right)}\right\|{\Psi_{2}J_{2}\rangle |}^{2},$$

and the excited state radiant lifetime can be described as

(6) $$\tau_{r}\left(\Psi_{1}J_{1}\right)=\frac{1}{\sum_{\Psi_{2}J_{2}}A_{r}(\Psi_{1}J_{1},\Psi_{2}J_{2})}.$$

Once the transition probability is obtained, the laser property-defining parameters can be estimated; the expression for the branching ratio is given as

(7) $$\beta_{r}(\Psi_{1}J_{1},\Psi_{2}J_{2})=\frac{A_{r}(\Psi_{1}J_{1},\Psi_{2}J_{2})}{\sum_{\Psi_{2}J_{2}}A_{r}(\Psi_{1}J_{1},\Psi_{2}J_{2})}.$$

Combining the UV–vis–NIR absorption spectra at room temperature and Equations (1)–(3), *f_exp_* is acquired and tabulated in Table 2. For *f_cal_*, the refractive indices of Sm:LN were measured to be *n* = 2.24 [2]. The values of *f_exp_* and *f_cal_*, shown in Table 2, indicate that Sm-1.0 possesses highest oscillator strengths for all the transitions compared with other samples. The value of Judd–Ofelt parameters, Ω_2_, Ω_4_, and Ω_6_, increased as the concentration of Sm^3+^ ions increased, and reached the max value at 1.0 mol.%, then decreased sharp at 2.0 mol.%; despite the change of Ω, all the samples have the same relation Ω_4_ > Ω_2_ > Ω_6_. Judd et al. [31] have reported that the hypersensitivenesses are associated with the large value of the reduced matrix elements ‖U^2^‖, and the hypersensitiveness is mainly described by the parameter Ω_2_. Compared with ‖U^2^‖, ‖U^4^‖ and ‖U^6^‖ have little effect on the hypersensitive transition. The hypersensitive degree of the rare-earth ion can be measured from the relative variation of the Ω_2_ for a rare-earth ion in different host environments. Table 2 shows the relation of the oscillator strength of the hypersensitive transition and Ω_2_, with the concentration of Sm^3+^ doped in Sm:LN. The values of Ω_2_ could be used to explain, with consideration of the peculiarity of the hypersensitive transition ^6^H_5/2_→^6^P_3/2_ in absorption spectra of Sm^3+^ ions, and the exceptionally large oscillator strength of the transition ^6^H_5/2_→^6^P_3/2_ changed with the Sm^3+^ concentration in Table 2, which clearly indicated the arising of the hypersensitive transition phenomenon. The value of hypersensitive transition ^6^H_5/2_→^6^P_3/2_ and Ω_2_, varies in a same manner as the Sm^3+^ concentration changed from 0.2 to 2.0 mol.%.

Next, the radiative transition rates and branching ratio for emission, from ^4^G_5/2_→^6^H_J_, were calculated at room temperature by using Equations (5)–(7); the results are displayed in Table 3. Obviously, the radiative transition rates and branching ratio of ^4^G_5/2_→^6^H_7/2_ and ^4^G_5/2_→^6^H_9/2_ transition are much higher than others, which is similar to that in Ref. [2]. The radiative lifetimes go down from 2465.54 to 1309.07 μs with the increasing of Sm^3+^ ion concentration from 0.2 to 1.0 mol.%, and then go up to 4480.89 μs when the concentration of Sm^3+^ ions reach 2.0 mol.%, which is similar to that of Sm^3+^-doped oxyfluoroborate glasses [32]. It is clear that the ^4^G_5/2_→^6^H_7/2_ transition, the orange-red radiative transition, was strengthened due to the increasing of doped Sm^3+^ ion concentration in the crystal. The “threshold concentration” of Sm^3+^-doped LN crystal is more superior in orange-red emission, which is quite useful for orange-red laser devices.

When compared to fluorozincate glass [14], oxyfluoride glass [17], silicate glass [33], PbFPSm10 and SNbKZFSm10 [34], the Sm (1.0 mol.%):LN single crystal possesses a higher value of Ω_2_, confirming the higher Sm–O covalency, and more asymmetry at the Sm^3+^ ion site. This result also indicates higher mixing of the opposite parity electronic configurations that are responsible for the spectral intensities. However, Ω_4_ and Ω_6_ are structure-dependent parameters that are related to the bulk properties of materials [14]. The spectroscopic quality factor, X, which is defined by the ratio of the intensity parameter Ω_4_ to Ω_6_ (Ω_4_/Ω_6_), is an important predictor for stimulated emission in a laser-active material. The spectroscopic quality factor X of Sm (1.0 mol.%):LN was calculated to be 2.66, that is larger than that of the other samples, suggesting that Sm (1.0 mol.%):LN single crystal is a promising laser material.

### 3.3. Fluorescence Analysis

**Figure 4:**
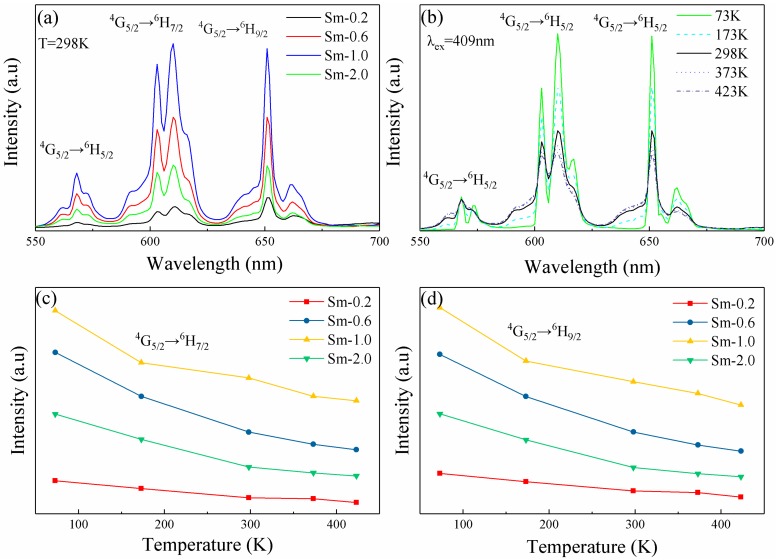
(**a**) The emission spectra of various concentration Sm:LN single crystals. (**b**) The emission spectra of 1.0 mol.% Sm:LN single crystals under series temperature. (**c**) The fluorescence intensity peaks of ^4^G_5/2_→^6^H_7/2_ corresponding to wavelength center 610 nm. (**d**) The fluorescence intensity peaks of ^4^G_5/2_→^6^H_9/2_ corresponding to wavelength center 651 nm.

Due to the strong absorption peak of the ^6^H_5/2_→^6^P_3/2_ transition, compared with that of other level transitions in the UV–vis region, a 409 nm laser is used to measure the emission spectra. Simultaneously, we use the area of the peaks in the NIR regions to calculate the spectral parameters. The fluorescence spectra of various Sm:LN single crystals were measured at room temperature, as shown in Figure 4a.

**Figure 5:**
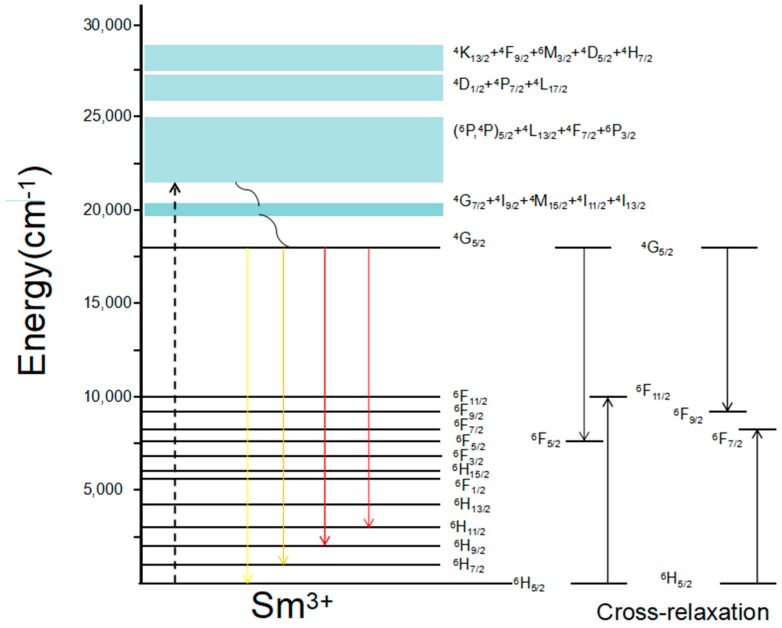
Simply energy levels diagram of Sm^3+^ ions in LN single crystals showing excitation and emission processes.

Figure 4a clearly shows that the luminescence intensity increases with the increasing concentration of Sm^3+^, and reaches a maximum at a concentration of 1.0 mol.% Sm^3+^. To study the emission spectra in more detail, we present a simple energy level transition diagram of the Sm^3+^ ion in Figure 5 that includes two energy transition patterns: a single ion of Sm^3+^ and cross-relaxation among multiple Sm^3+^ ions [14].

There are main three transitions under 409 nm laser excitation corresponding to ^4^G_5/2_→^6^H*_J_* (*J* = 5/2, 7/2, 9/2) in Figure 5. Based on the level energies of the samarium ion, we found the transitions ^4^G_5/2_→^6^F_11/2_ = ^6^H_5/2_→^6^F_5/2_, ^4^G_5/2_→^6^F_5/2_ = ^6^H_5/2_→^6^F_11/2_ occur in the crystals, and indicate a cross-relaxation process, such as with ^4^G_5/2_→^6^F_9/2_ ≈ ^6^H_5/2_→^6^F_7/2_ and ^4^G_5/2_→^6^F_7/2_ ≈ ^6^F_9/2_, as shown in Figure 5. This transition mechanism may explain the relationship between the concentration and fluorescence intensity.

Sm^3+^ ions have two energy transition patterns: (1) when the concentration of Sm^3+^ in the crystal is less than 1 mol.%, the radiation processes of Sm^3+^ are independent; i.e., the luminescence intensity enhances as the concentration increases; (2) when the concentration of Sm^3+^ exceeds the threshold value, the emission spectra mainly rely on energy transfer between Sm^3+^ ions; i.e., the emission spectra of Sm^3+^ is primarily caused by the increased energy transfer between Sm^3+^ ions via nonradioactive processes in heavily Sm^3+^-doped LiNbO_3_ single crystals. The prominent emission spectral peaks at 610 and 651 nm may be attributed to the multiphonon-assisted nonradiative relaxation in the crystal [34]. Hence, we have determined the relationship between Sm^3+^ and fluorescence intensity, and the mechanism of the energy level transitions of Sm^3+^. This mechanism can explain the fluorescence intensity of 2.0 mol.% Sm:LN being weaker than that of 1.0 mol.% Sm:LN well.

To investigate the effect of temperature on the fluorescence property of the Sm^3+^ ions in Sm:LN, we selected the 1.0 mol.% Sm:LN sample, and measured the fluorescence spectra under 409 nm laser excitation as the temperature changed from 73 to 423 K, as shown in Figure 4b. We found that the peak intensities of all samples decreased with the temperature increase, and the split peak shoulders of ^4^G_5/2_→^6^H_7/2_ and ^4^G_5/2_→^6^H_9/2_ are more apparent at a lower temperature than a higher temperature in the emission spectra at 610 nm [35]. Figure 4c shows the fluorescence intensity peak of ^4^G_5/2_→^6^H_7/2_, corresponding to the center of the peak at 610 nm. Figure 4d shows the fluorescence intensity peak of ^4^G_5/2_→^6^H_9/2_, corresponding to the center of the peak at 651 nm. From Figure 4c,d), it is shown that concentration of Sm^3+^ ions has great effect on the fluorescence intensity. The fluorescence intensity of Sm (1.0 mol.%):LN is 10 times and 13 times that in Sm (0.2 mol.%):LN at 610 nm and 651 nm, respectively. We can see that all the magnitudes of the fluorescent peaks are maximized when the concentration of Sm^3+^ is 1.0 mol.%, and the peaks for each concentration of Sm:LN crystals follow the same rule; i.e., the fluorescent peaks gradually decrease as the temperature increases. The reason for this behavior is the cross-relaxation process of Sm^3+^ ions, as shown in Figure 5, indicating that multiphonon relaxation of the ^4^G_5/2_ level plays an important role in the measured fluorescent spectra.

**Figure 6:**
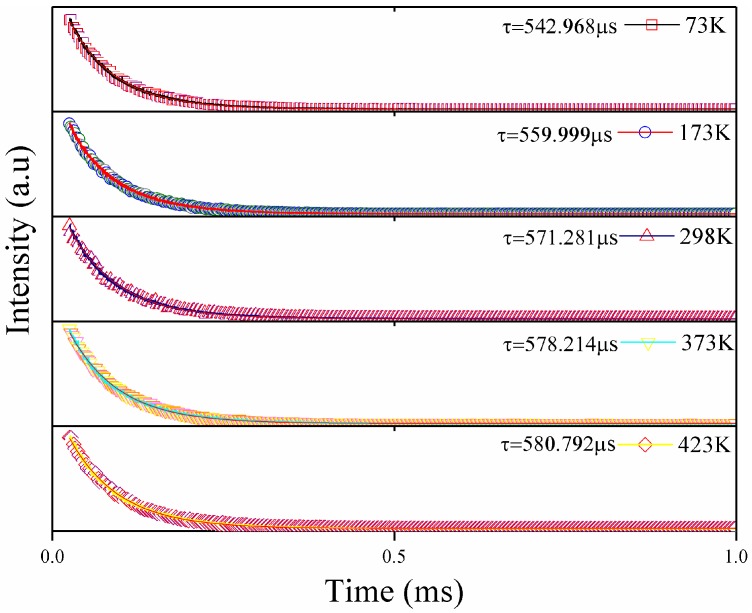
The fluorescence decay curves of the ^4^G_5/2_→^6^H_9/2_ transition in 1.0 mol.% Sm:LN single crystal at different temperatures.

The experimental lifetimes of 1.0 mol.% Sm:LN were measured by monitoring the emission at 651 nm, corresponding to the ^4^G_5/2_→^6^H_9/2_ transition upon 409 nm pulsed laser excitation at various temperatures, as presented in Figure 6. The lifetime was found to gradually increase with the increasing temperature. However, the maximum change is only approximately 40 µs, which indicated that the lifetime of Sm^3+^ in Sm:LN single crystals is not sensitive to temperature.

The stimulated emission cross-section (*σ_em_*) is an important parameter for predicting the energy extraction efficiency of a laser material. The stimulated emission cross-sections, *σ_em_*, can be calculated using the formula

*σ_em_* = *λ*^5^*βI*(*λ*)/(8*πcn*^2^*τ_r_∫λI*(*λ*)*dλ*), (8)

where *I*(*λ*) is the experimental emission intensity as a function of the peak position, *c* is the light velocity, *n* is the mean refractive index, *β* is the branching ration of the transition, and *τ_r_* is the radiative lifetime of the fluorescence level. The values of *β* and *τ_r_* were calculated earlier in this paper using the Judd–Ofelt method. For Sm (1.0 mol.%):LN, the transitions of ^4^G_5/2_→^6^H_7/2_, ^4^G_5/2_→^6^H_7/2_, and ^4^G_5/2_→^6^H_9/2_ correspond to the center of the peaks at 568, 610, and 651 nm, respectively. The corresponding stimulated emission cross-sections, *σ_em_*, of the peaks at 568, 610, and 651 nm, are 0.32 × 10^−20^ cm^2^, 1.257 × 10^−20^ cm^2^, and 1.081 × 10^−20^ cm^2^, respectively. Those values are much higher than the stimulated emission cross-section in the glass materials, such as Sm^3+^ in fluoroborate glasses, LKBBT, PbFPSm10, PKBASm10, PKBFASm10, and oxyfluoroborate [2,14,15,16,17,36].

The higher branching ratios and stimulated emission cross-sections for the ^4^G_5/2_→^6^H_7/2_ and ^4^G_5/2_→^6^H_9/2_ transitions suggest that the Sm:LN single crystals can be useful for laser applications. The higher stimulated emission cross-section is also favorable for low threshold and high gain laser applications, and can be utilized to obtain continuous-wave laser action [36]. Since the Sm:LN single crystals exhibit larger stimulated emission cross-sections and branching ratios, they are suitable for use in the development of visible lasers and optical fiber amplifiers.

## 4. Conclusions

Congruent Sm:LN single crystals, with concentrations of trivalent samarium ions changing from 0.2 to 2.0 mol.%, were grown using the Czochralski method. Based on XRD data and UV absorption spectra, the locations of the Sm^3+^ ions and the shifts in the absorption edges of Sm:LN were analyzed; after examining the emission spectra, we found that the optimal concentration of Sm^3+^ ions is 1.0 mol.%. The ${\mathrm{Sm}}_{\mathrm{Li}}^{2+}$${–\mathrm{Sm}}_{\mathrm{Nb}}^{2-}$ centers existed in highly doped Sm:LN single crystals, and play an important role in the process of luminescence. The fluorescence spectra obtained over a large temperature range demonstrated that the fluorescence intensity decreased with increasing temperature. However, the lifetime of the transition ^4^G_5/2_→^6^H_9/2_ only changed slightly with the increasing temperature. This observation indicates that the lifetime is not sensitive to the working temperature. Cross-relaxation among Sm^3+^ ions strongly depends on the material composition, because the lifetime of Sm^3+^ ions is very different in different materials. Under 409 nm laser excitation, the visible fluorescence spectrum has green light at 568 nm, orange bands at 610 nm, and reddish-orange light at 651 nm. The fluorescence branching ratio, *β*, for the^4^G_5/2_→^6^H_7/2_ and ^4^G_5/2_→^6^H_9/2_ transitions, is 0.4347 and 0.3846, respectively, indicating that Sm:LN single crystals can be an attractive laser material to exhibit efficient visible lasing emissions in the orange spectral region. We can adjust and control the ratio of the orange-red light through the concentration of Sm^3+^ ions and the environmental temperature. Such control also provides a method to regulate and control spectral components in visible-light region.

## Figures and Tables

**Table 1:** The lattice constants of the crystal samples.

Sample	*a* (Å)	*b* (Å)	*c* (Å)	*V* (Å^3^)	*c*/*a*
CLN	5.12157	5.12157	13.80294	313.55	2.69348
Sm-0.2	5.14798	5.14798	13.85091	317.89	2.69055
Sm-0.6	5.15059	5.15059	13.84371	318.05	2.68779
Sm-1.0	5.15322	5.15322	13.84783	318.47	2.68722
Sm-2.0	5.15972	5.15972	13.85047	319.34	2.68435

**Table 2:** Experimental and calculated oscillator strengths (10^−6^) for Sm:LN and the Judd–Ofelt intensity parameters Ω_λ_ (10^−20^), the root mean square deviation △δ_rms_, and the spectroscopic quality factor *X* (Ω_4_/Ω_6_).

Transition ^6^H_5/2_→	Energy (cm^−1^)	Oscillator Strengths							
		Sm-0.2		Sm-0.6		Sm-1.0		Sm-2.0	
		*f_exp_*	*f_cal_*	*f_exp_*	*f_cal_*	*f_exp_*	*f_cal_*	*f_exp_*	*f_cal_*
^4^D_3/2_	27,322	-	-	-	-	1.20	1.45	-	-
^6^P_3/2_, ^6^P_5/2_	24,150	4.86	4.89	5.04	5.31	9.31	9.99	2.90	2.93
^4^I_13/2_, ^4^I_11/2_	20,833	1.15	0.44	1.56	0.46	2.60	2.01	0.89	0.27
^6^F_11/2_	10,471	0.34	0.29	0.36	0.30	0.57	0.88	0.14	0.18
^6^F_9/2_	9152	1.99	1.87	1.98	1.92	4.50	2.91	1.14	1.14
^6^F_7/2_	7964	2.45	2.64	2.47	3.16	5.00	5.16	1.35	1.83
^6^F_5/2_, ^6^F_3/2_, ^6^F_1/2_, ^6^H_15/2_	6680	3.38	2.89	3.14	3.15	6.03	5.38	1.61	1.74
		Ω_2_ = 1.39		Ω_2_ = 1.52		Ω_2_ = 2.37		Ω_2_ = 0.84	
		Ω_4_ = 2.36		Ω_4_ = 2.56		Ω_4_ = 4.82		Ω_4_ = 1.41	
		Ω_6_ = 1.21		Ω_6_ = 1.24		Ω_6_ = 1.81		Ω_6_ = 0.74	
		X = 1.95		X = 2.06		X = 2.66		X = 1.91	
		△δ_rms_ = 0.52		△δ_rms_ = 0.79		△δ_rms_ = 0.99		△δ_rms_ = 0.46	

**Table 3:** The peak wavelength (λ*_em_*), radiative transition probability (*A_r_*), branching ratio (*β*), total radiative transition probability (∑*A_r_*), and radiative lifetime (*τ_r_*).

Transition ^4^G_5/2_→	*λ_em_*	Sm-0.2		Sm-0.6		Sm-1.0		Sm-2.0	
	(nm)	A(S^−1^)	*β*(%)	A(S^−1^)	*β*(%)	A(S^−1^)	*β*(%)	A(S^−1^)	*β*(%)
^6^H_5/2_	568	15.51	0.0382	16.85	0.0387	30.93	0.0405	9.29	0.0416
^6^H_7/2_	610	178.34	0.4397	189.92	0.4360	332.05	0.4347	86.72	0.3886
^6^H_9/2_	651	160.38	0.3954	173.86	0.3991	296.81	0.3846	96.48	0.4323
^6^H_11/2_	717	51.36	0.1266	54.96	0.1262	104.11	0.1363	30.68	0.1375
∑A_r_(S^−1^)		405.59		435.59		763.90		223.17	
*τ*_r_ (μs)		2465.54		2295.74		1309.07		4480.89	
